# A randomized cross-over trial to detect differences in arm volume after low- and heavy-load resistance exercise among patients receiving adjuvant chemotherapy for breast cancer at risk for arm lymphedema: study protocol

**DOI:** 10.1186/s12885-016-2548-y

**Published:** 2016-07-22

**Authors:** Kira Bloomquist, Sandi Hayes, Lis Adamsen, Tom Møller, Karl Bach Christensen, Bent Ejlertsen, Peter Oturai

**Affiliations:** University Hospitals Centre for Health Research (UCSF), Copenhagen University Hospital, Rigshospitalet, Blegdamsvej 9, 2100 Copenhagen Ø, Denmark; Institute of Health and Biomedical Innovation, Queensland University of Technology, 60 Musk Avenue, Kelvin Grove Urban Village, Kelvin Grove, Queensland 4059 Australia; Department of Public Health; Section of Biostatistics, University of Copenhagen, Øster Farimagsgade 5, 1014 Copenhagen K, Denmark; DBCG, Afsnit 2501, Copenhagen University Hospital, Blegdamsvej 9, 2100 Copenhagen Ø, Denmark; Department of Clinical Physiology, Nuclear Medicine and PET, Copenhagen University Hospital, Blegdamsvej 9, 2100 Copenhagen, Denmark

**Keywords:** Lymphedema, Breast cancer, Resistance exercise

## Abstract

**Background:**

In an effort to reduce the risk of breast cancer-related arm lymphedema, patients are commonly advised to avoid heavy lifting, impacting activities of daily living and resistance exercise prescription. This advice lacks evidence, with no prospective studies investigating arm volume changes after resistance exercise with heavy loads in this population. The purpose of this study is to determine acute changes in arm volume after a session of low- and heavy-load resistance exercise among women undergoing adjuvant chemotherapy for breast cancer at risk for arm lymphedema.

**Methods/Design:**

This is a randomized cross-over trial. *Participants*: Women receiving adjuvant chemotherapy for breast cancer who have undergone axillary lymph node dissection will be recruited from rehabilitation centers in the Copenhagen area. *Intervention:* Participants will be randomly assigned to engage in a low- (two sets of 15–20 repetition maximum) and heavy-load (three sets of 5–8 repetition maximum) upper-extremity resistance exercise session with a one week wash-out period between sessions. *Outcome:* Changes in extracellular fluid (L-Dex score) and arm volume (ml) will be assessed using bioimpedance spectroscopy and dual-energy x-ray absorptiometry, respectively. Symptom severity related to arm lymphedema will be determined using a visual analogue scale (heaviness, swelling, pain, tightness). Measurements will be taken immediately pre- and post-exercise, and 24- and 72-hours post-exercise. *Sample size:* A sample size of 20 participants was calculated based on changes in L-Dex scores between baseline and 72-hours post exercise sessions.

**Discussion:**

Findings from this study are relevant for exercise prescription guidelines, as well as recommendations regarding participating in activities of daily living for women following surgery for breast cancer and who may be at risk of developing arm lymphedema.

**Trial registration:**

Current Controlled Trials ISRCTN97332727. Registered 12 February 2015.

**Electronic supplementary material:**

The online version of this article (doi:10.1186/s12885-016-2548-y) contains supplementary material, which is available to authorized users.

## Background

Approximately 20 % of breast cancer survivors develop breast cancer-related arm lymphedema BCRL [1], with an estimated 80 % of cases presenting within the first two years of diagnosis [2]. It is associated with significant impairments in gross and fine motor skills affecting work, home and personal care functions, as well as recreational and social relationships [3, 4]. While the etiology of BCRL is unknown [1, 5], findings from a systematic review and meta-analysis from 2013 [1] including 72 studies demonstrate that axillary lymph node dissection, more extensive breast surgery, radiotherapy, chemotherapy, being overweight or obese and physical inactivity are consistently associated with increased BCRL risk [1].

Participation in resistance exercise has been found to be a safe and effective exercise modality among breast cancer survivors at risk of BCRL [6, 7], and is associated with increases in lean muscle mass and strength, which in turn positively effect physical function and ability. Furthermore, findings from a recent meta-analysis [6] suggest that resistance exercise can reduce the risk of BCRL versus control conditions (OR = 0.53 (95 % CI 0.31–0.91); I^2^ = 0 %). However, the current evidence-base is derived from studies that have evaluated resistance exercise intensities considered to be low to moderately heavy (60–80 % of 1 repetition maximum (RM) or 8–15 RM) [6, 7]. Yet, exercise science literature indicates that heavy-load resistance exercise (80–90 % 1RM or 5–8 RM) [8] is more effective than low- to moderate-load resistance exercise in generating muscle strength gains [9]. There is therefore a clear need for studies evaluating the safety of heavy-load resistance exercise in the at-risk population [7].

In a novel study by Cormie et al. [10], which evaluated the effect of low- and heavy-load resistance exercise among a sample with BCRL, lymphedema status and lymphedema symptoms remained stable immediately after exercise, and 24- and 72-hours after exercise, irrespective of load. While these findings provide important information for women with BCRL, the purpose of this study is to determine acute changes in extracellular fluid, arm volume and associated lymphedema symptoms after a session of low- and heavy-load resistance exercise in women at risk for BCRL. It is hypothesized that no interlimb differences in extracellular fluid, arm volume or lymphedema-associated symptom severity will be observed over time or between resistance exercise loads.

## Design

This study is a randomized, cross-over trial (Table 1 here).

**Table 1:** Trial registration data

Trial registration data	
Primary registry and trial identify number	Current Controlled Trials ISRCTN97332727.
Date of registration in primary registry	12 February 2015.
Secondary identifying numbers	H-3-2014-147, 30-1430
Source of monetary or material support	University Hospitals Centre for Health Research, Copenhagen University Hospital Rigshospitalet
Primary sponsor	University Hospitals Centre for Health Research, Copenhagen University Hospital Rigshospitalet
Secondary sponsor	
Contact for public queries	KB, MHS, PhD-stud. kibl30@hotmail.com, (45) 35347362, Blegdamsvej 9 (afsnit 9701), 2100 Copenhagen
Contract for scientific queries	KB, MHS, PhD-stud. kibl30@hotmail.com, (45) 35347362, Blegdamsvej 9 (afsnit 9701), 2100 Copenhagen
Public title	A trial to detect differences in arm volume after low- and heavy-load resistance exercise among patients receiving adjuvant chemotherapy for breast cancer at risk for arm lymphedema: Study Protocol
Scientific title	A randomized cross-over trial to detect differences in arm volume after low- and heavy-load resistance exercise among patients receiving adjuvant chemotherapy for breast cancer at risk for arm lymphedema: Study Protocol
Countries of recruitment	Denmark
Health condition or problem studied	Breast cancer-related arm lymphedema
Intervention	Heavy vs low load resistance exercise for the upper extremities
Key inclusion and exclusion criteria	Inclusion criteria: > 18 years of age, unilateral breast surgery, axillary node dissection, undergoing adjuvant chemotherapy for breast cancer
	Exclusion criteria: Previously treated for breast cancer, diagnosis of BCRL and/or currently receiving treatment for BCRL, or having conditions hampering resistance exercise of the upper body, or having participated in regular upper-body heavy resistance exercise during the last month
Study type	Interventional
	Randomized cross-over, assessor blinded
	Safety
Date of first enrolment	31-03-2015
Target sample size	40
Recruitment status	Recruiting
Primary outcome	Arm extracellular fluid (L-dex score) post-, 24- and 72 h post exercise
Key secondary outcomes	Arm volume (ml) post-, 24- and 72 h post exercise

## Methods

### Participants / Recruitment

**Fig. 1:**
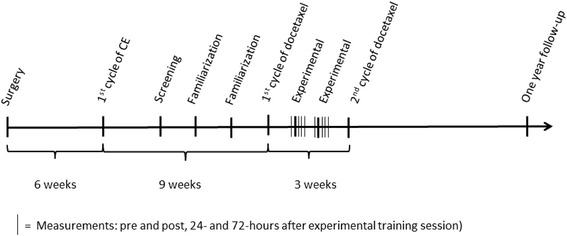
Study time line

Twenty women allocated to adjuvant chemotherapy for breast cancer consisting of three cycles of 3-weekly epirubicin followed by three cyles of 3-weekly docetaxel will be recruited from municipality lead rehabilitation centers in the Copenhagen area and from a waiting list to the *Body and Cancer* program [11, 12], at the University Hospitals Center for Health Research (UCSF) at the Copenhagen University Hospital, Rigshospitalet. All patients will be screened for inclusion by health professionals (nurse or physical therapist) at the respective centers. Potential participants fulfilling inclusion criteria; over 18 years of age, unilateral breast surgery, axillary node dissection, and initiating /undergoing adjuvant chemotherapy for breast cancer (stage I - III) will be contacted during their first three cycles of chemotherapy (Fig. 1). Patients previously treated for breast cancer, with a diagnosis of BCRL and/or currently receiving treatment for lymphedema, or having conditions hampering resistance exercise of the upper body, or having participated in regular (>1 × / week) upper-body heavy resistance exercise during the last month will be excluded.

Those fulfilling study criteria and expressing interest in study participation will thereafter be screened for BCRL by the first author after the third cycle of chemotherapy, using bioimpedance spectroscopy (BIS). Furthermore, in accordance with common toxicity criteria (CTC) v3.0 lymphedema criteria for the limb [13], patients will be visually inspected to detect differences in signs of swelling between arms. Those presenting with BCRL, defined as a lymphedema index (L-Dex) score of 10 or greater [14–16] (as assessed by BIS), and/or visual signs of swelling (obscuration of anatomic architecture or pitting edema) of the at-risk arm [13] will be referred for treatment, and will not be included in the study.

Written and oral information regarding the study will be delivered by the first author, as well as obtainment of informed written consent.

### Concealed randomization

Prior to the study, a computer-generated random sequence will be generated by an external researcher not otherwise affiliated with the study, and concealed in opaque envelopes. Group assignment will be disclosed to the first author by telephone after study inclusion and participation in the familiarization period. Participants will be allocated using a 1:1 ratio to partake in either low- or heavy-load resistance exercise first.

### Exercise sessions

Participants will engage in a familiarization period, comprising of two training sessions up to one week apart, after the third cycle of chemotherapy. Each session will start with a 10- minute aerobic warm-up using a cross-trainer (Glidex, Technogym®, Gamettola, Italy). During the first familiarization session participants will be introduced to four upper-body exercises (chest press, latissimus pull down, triceps extension (Technogym®, Gamettola, Italy) and biceps curl (free weights)) followed by a 1RM strength test in each exercise. At the second familiarization session, two sets of 10–15 RM will be performed and a new 1RM strength test will be undertaken to ensure accuracy of subsequent exercise prescription. Participants will engage in the first experimental session after the first cycle of docetaxel (fourth chemotherapy), followed by a wash-out period of 6 days. Two sets of 15–20 RM of each exercise will be performed during low-load resistance exercise and three sets of 5–8 RM during heavy-load. All sets will be performed to muscle fatigue in sessions individually supervised by the first author (a physical therapist with experience in exercise prescription for women with breast cancer) at training facilities located at Rigshospitalet.

### Outcomes (pre- and post, 24- and 72-hours after resistance exercise)

Measurements will be performed by medical technicians with no knowledge of group (low- / high-load first) allocation at the Department of Clinical Physiology and Nuclear Medicine at the Copenhagen University Hospital, Rigshospitalet. Participants are advised to maintain their normal activities during study participation. At all assessment points, participants will be asked about their physical activities, and any extraordinary activities will be recorded.

#### Primary outcome

*Extracellular fluid* BIS (SFB7, Impedimed, Brisbane, Australia) directly measures the impedance of extracellular fluid and has a high reliability for detecting BCRL [14, 16, 17] (intraclass correlation coefficient (ICC) = 0,99) [18]. Participants will be positioned in supine with arms and legs slightly abducted from the trunk with palms facing down. Utilizing the principle of equipotentials, four single tab electrodes will be placed in a tetrapolar arrangement [17]. Measurement electrodes will be placed on the dorsum of the wrist midway between the styloid processes, with current drive electrodes placed five centimeters distally on the dorsal side over the third metacarpal of the hand, and approximately midway on the third metatarsal on the dorsum of the foot [17, 19]. Each limb will be measured at a range of frequencies using the manufacturer’s software. The ratio of impedance between the at-risk and non-affected limb will be calculated and converted into a L-Dex score.

#### Secondary outcomes

*Arm volume* Dual energy x-ray absorptiometry (DXA) (Lunar Prodigy Advanced Scanner, GE Healthcare, Madison, WI) measures tissue composition using a three-compartment model that is sensitive to changes in upper-limb tissue composition [20, 21]. Using previously derived densities for: fat (0.9 g/ml); lean mass (1.1 g/ml); bone mineral content (BMC) (1.85 g/ml), the measured DXA tissue weights will be transformed into estimated arm volumes [20, 21].

**Fig. 2:**
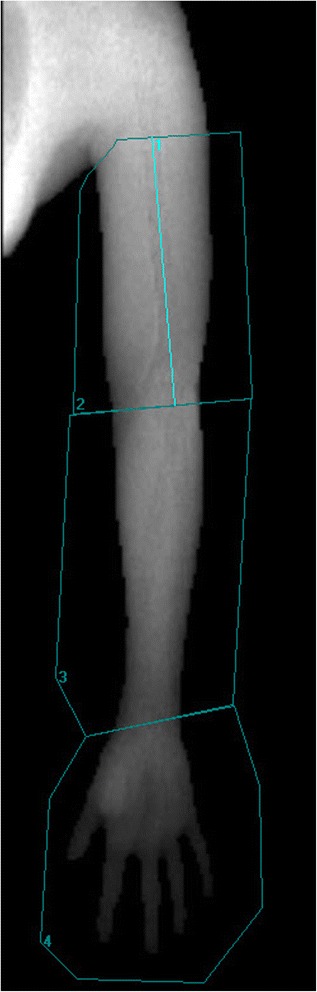
DXA regions of interest

Participants will be positioned on the scan-table, lying supine with the arm separated from the trunk. If necessary a Velcro band will be used over the breast to ensure space between the arm and truncus. Each arm will be scanned separately. Small animal software (Encore version 14.10) will be used to analyze the scans as described by Gjorup et al. [20]. Scans will be point typed where soft tissue is marked as bone, whereafter regions of interest (ROIs) will be drawn around the hand and the arm on every scan (Fig. 2). All scans will be analyzed by one examiner (last author) with experience in analyzing DXA scans.

*Subjective assessment of symptoms* The severity of symptoms related to arm lymphedema including swelling, heaviness, pain and tightness, will be monitored using a visual analogue scale, whereby 0 represents no discomfort and 10 is indicative of very severe discomfort [10].

### One year follow-up

Statistically, it is assumed that some of the participants in the study will develop arm lymphedema. Furthermore, previous studies have found a highly variable response to resistance exercise [10, 22]. A one year exploratory, hypothesis generating follow-up has been planned as it provides an opportunity to determine how many participants develop arm lymphedema and whether individual variability in response to the resistance exercise sessions is related to subsequent lymphedema incidence. Measurements will include 1RM strength in the four resistance exercises, DXA, BIS and symptom severity (VAS) as described, and a structured interview by the first author to determine other known and theoretical risk factors.

### Blinding

All data collection and analysis will be conducted by study personnel with no knowledge of group (low- / high-load first) allocation.

### Sample size and analytical plan

The sample size calculation is based on changes in L-Dex scores between baseline and 72 h post-resistance exercise sessions. From results of Cormie et al. [10] we hypothesize the standard deviation in the distribution to be 1.9 units. No published normative change scores exist for the at-risk population, as well as no evidence regarding a threshold for a clinically significant acute change. For patients with BCRL a change score of 2L-Dex units is considered clinically relevant based on clinical experience. We believe that an L-Dex of 2 units is too conservative in the at-risk population, and have therefore set a threshold at 3L-Dex units. Thus, if there is no difference between groups, then 18 patients are required to be 90 % sure that the limits of a two-sided 90 % confidence interval will exclude a difference in means of more than 3.0. To allow for possible drop-outs we plan to include 20 patients.

Data will be analyzed using the Statistical Package for Social Sciences (SPSS) software (version 19) for Windows (IBM SPSS, Chicago, IL). Analysis will include standard descriptive statistics and both intention to treat and per-protocol analysis will be performed. Using a generalized estimating equations framework for continuous outcomes to determine time (baseline, pre-, post, 24- and 72 h) and intervention (low-/ heavy-load) effects, the interaction between time and intervention will be considered [23]. Two-tailed *p* < 0.05 will be taken as evidence of statistical significance.

### Safety and ethical considerations

The treating oncologist will have the overall responsibility for the participants. All personal data will be treated in accordance with existing rules and regulations.

A full body DXA scan utilizes weak x-rays and is not considered dangerous [24]. In this study, since only arms will be scanned the radiation dose is estimated to be 0.0001 mSv for both arms. Eight scans result in a total dose of 0.0008 mSv, which is less than the background radiation an average person is exposed to in one day in Denmark.

As about 20 % of women treated for breast cancer develop BCRL [1], it is expected that some of the participants in this study will develop BCRL. Participation in this study involves regular assessment of the at-risk arm during the study period, using some of the best technology to date. This allows for early detection of BCRL, which in turn would render a better prognosis, as early detection is associated with a better outcome [4]. If participants develop signs of swelling or an L-Dex score persisting over one week during the familiarization or experimental study period, they will be referred to the treating oncologist for lymphedema treatment and will be withdrawn from the study.

A completed SPIRIT checklist is included as Additional file 1.

## Discussion

Participating in resistance exercise during adjuvant chemotherapy for breast cancer has been associated with increases in muscle strength [25–29], lean body mass [25, 28], and self-esteem [25], and has been found to mitigate fatigue and to maintain quality of life [29]. Furthermore, there is evidence to suggest that resistance exercise might be associated with a higher completion rate of planned chemotherapy [25]. Moreover, generalized edema characterized by an increase in the size of the interstitial compartment of extracellular fluid is a potential side effect to taxane-based chemotherapy [30, 31]. Thus, swelling as a consequence of increased fluid in addition to an impairment of lymph fluid transport, could potentially contribute to swelling of the at-risk arm. Hypothetically, this could be thwarted by resistance exercise due to increased lymph clearance likely through the effects of the muscle pump [32, 33], lending additional rationale for instigating resistance exercise during adjuvant chemotherapy.

To our knowledge, studies investigating the safety and efficacy of resistance exercise in patients at risk for BCRL have utilized low- to moderate-resistance exercise intensities [6, 7], with only one cross-sectional study [11] investigating heavy-load resistance exercise. Indeed, in a paper identifying the top 10 research questions related to physical activity and cancer survivorship, Courneya et al. [34] highlighted the need for studies investigating safety and optimal exercise prescription, and specifically the role of vigorous-intensity activity, as important research areas [34].

The rational for utilizing heavy-load resistance exercise is supported by exercise science literature that indicates that this higher training intensity can lead to additional benefits as a dose–response relationship exists between the load of resistance exercise and gains in muscular structure and function [35, 36]. Furthermore, breast cancer survivors may suffer from losses of bone mass (particularly those on aromatase inhibitors), at least in part as a result of the catabolic effects of treatment. Resistance exercise interventions with lower loads have not yielded significant training effects on bone mineral density [27, 37]. It has been postulated that the absence of a measurable effect on bone mass density is related to the adaptive nature of bone that requires heavier loads [37], as heavy-load resistance exercise has been identified as an osteogenic exercise modality in women without cancer [38]. Thus, establishing the safety of heavy-load resistance exercise is prudent and of significance for the breast cancer population.

No standardized measurement method exists to diagnose or monitor BCRL [1, 20, 21], with a variety of techniques and definitions used. Early BCRL is characterized by an increase in extracellular fluid. Indirect measurement methods such as circumference, water displacement, and perometry measure volume of the entire limb to detect small changes in extracellular fluid which accounts for approximately 25 % of the total limb, and do not differentiate between tissue types [5, 18]. In contrast, BIS directly measures lymph fluid change by measuring the impedance to a low level electrical current allowing for a sensitive [21, 39] and reliable measurement method to detect extracellular fluid changes among at-risk breast cancer survivors [39]. Furthermore, BIS is fast and easy to administer, and as impedance measures are reported as an L-Dex value, inherent volume differences associated with hand dominance are taken into account [5, 39]. However, BIS loses its sensitivity to monitor BCRL over time as lymphedema progresses into later stages, whereby the excess extracellular fluid initially characterizing BCRL is replaced with adipose tissue [5, 21].

DXA is another measurement method that can differentiate between tissue types giving an estimate of BMC, fat mass and lean mass where the lean mass component includes extracellular fluid [20, 21, 40]. DXA has been found to be sensitive to changes in tissue composition, making it an ideal measurement method to monitor BCRL over time as fluid components are replaced with adipose tissue. Furthermore, DXA allows for analysis of separate regions of the arm, of potential clinical importance for patients where swelling is confined to a specific region of the arm or hand [20, 21, 40, 41]. In this study we scan the arms separately and use software with a high resolution allowing for more precise definition of ROIs and the possibility to define bone and soft tissue manually as described by Gjorup et al., with a low inter-rater variation (ICC ≥,9990) [20]. To the authors’ knowledge, this is the first time that DXA, with this software, will be used to detect volume changes in the BCRL at-risk population adding new insights into the application of this measurement method.

This exploratory study utilizes a cross-over design to determine acute changes in extracellular fluid and arm volume. This design lends more statistical power, with the practical advantage of a smaller sample size, as between-patient variation is inherently eliminated [42], providing a framework for an efficient comparison between the two resistance exercise loads. However, this study can only provide us with information regarding extracellular fluid and arm volume changes after one resistance exercise session, limiting the generalizability to repeated resistance exercise training and long-term effects on arm volume. Nonetheless, this study can lend initial evidence regarding the safety of heavy-load lifting and can help guide future studies and optimal exercise prescription.

Finally, women at risk for BCRL still receive risk reduction advice including avoiding heavy lifting [43, 44]. This advice can lead to women being apprehensive about lifting heavy loads with consequences for daily living (e.g., not lifting children, groceries, etc.). However, this advice is not based on research and knowledge gained from this study can provide a preliminary evidence base for guiding risk reduction practices involving intermittent heavy-load activity necessary for daily living.

## Abbreviations

AND, axillary lymph node dissection; BCRL, breast cancer-related arm lymphedema; BIS, bioimpedance spectroscopy; CTC, common toxicity criteria; DXA, dual-energy x-ray absorptiometry; ICC, intraclass correlation coefficient; L-Dex, lymphedema index; ROI, region of interest

## Additional file

Additional file 1: SPIRIT checklist. (DOC 121 kb)
